# Survival after parotid gland metastases of cutaneous squamous cell carcinoma of the head and neck

**DOI:** 10.1007/s10006-020-00934-8

**Published:** 2021-01-05

**Authors:** Moritz Friedo Meyer, Philipp Wolber, Christoph Arolt, Maximilian Wessel, Alexander Quaas, Stephan Lang, Jens Peter Klussmann, Robert Semrau, Dirk Beutner

**Affiliations:** 1 Department of Otorhinolaryngology, Head and Neck Surgery, University Hospital Essen, University Duisburg-Essen, Hufelandstraße 55, 45122 Essen, Germany; 2 Department of Otorhinolaryngology, Head and Neck Surgery, Medical Faculty, University of Cologne, Cologne, Germany; 3 Department of Pathology, University Hospital of Cologne, Cologne, Germany; 4 Radiotherapy Bonn-Rhein-Sieg, Troisdorf, Germany; 5 Department of Otorhinolaryngology, Head and Neck Surgery, University Medical Center Göttingen, Göttingen, Germany

**Keywords:** Salivary gland carcinoma, Therapy, Survival, Cutaneous squamous cell carcinoma, Skin, Parotid

## Abstract

**Purpose:**

Malignant tumours in the parotid gland can originate either from the gland itself or as a result of metastatic spread of other tumours, such as cutaneous squamous cell carcinomas (CSCC) of the head and neck area. The aim of this study was to analyse and compare the clinical behaviour of primary as well as CSCC metastatic parotid cancers with special emphasis on therapy and oncologic outcome.

**Methods:**

Clinical and histopathological data of 342 patients with parotid gland malignomas surgically treated in a tertiary referral centre between 1987 and 2015 were retrospectively assessed. Oncologic outcomes of all cases with CSCC metastasis of the parotid gland (*n* = 49) were compared to those of primary parotid gland carcinomas (*n* = 293).

**Results:**

Mean age at diagnosis was 72.3 years for CSCC patients versus 56.8 years in patients with primary parotid carcinoma. A total of 83.7% of CSCC patients were male, compared to 48.8% in the group of primary carcinomas. Forty-five out of 49 CSCC patients underwent total parotidectomy and neck dissection (91.8%). A total of 93.9% out of all CSCC patients received adjuvant radiotherapy. Five-year overall survival (OS) was 32.6% in CSCC patients versus 77.2% in primary parotid carcinoma patients.

**Conclusion:**

As compared to primary parotid cancers, we could show that patients suffering from CSCC metastases to the parotid gland presented with significantly higher age and worse survival.

## Introduction

Salivary gland carcinomas (SGC) account for less than 1% of all cancer types in Europe [1]. SGC are most frequently localised in the parotid gland, although the proportion of malignant to benign tumours in the small salivary glands is higher [2]. According to the huge diversity of tumour subtypes and the low incidence, appropriate treatment remains challenging. Twenty subtypes of SGC have been defined by the World Health Organisation yielding different histological and molecular characteristics [3]. Mucoepidermoid carcinoma is the most common subtype [4, 5].

Due to possible facial nerve involvement, parotid gland carcinomas (PGC) can be challenging for head and neck surgeons. The biological aggressiveness of PGC varies considerably between the different entities. For example, the overall survival ranges between 95–100% for low-grade adenocarcinoma [6] and 23–50% in high-grade mucoepidermoid carcinoma cases [7]. Prognosis is significantly impaired by loco-regional lymph node metastases [4].

Complete tumour removal (R0) is the most effective treatment for PGC. Elective treatment of the N0 neck remains a controversial issue. Radiotherapy can be used as adjuvant therapy in patients with risk factors [2].

Squamous cell carcinomas (SCC) of the parotid gland have a worse prognosis as compared to other malignant tumours of the parotid gland, such as adenoid cystic, mucoepidermoid, and acinic cell carcinomas [8]. Tumorigenesis of squamous cell carcinoma of the parotid gland [9] is still under discussion: While some might consider primary SCC of the salivary glands as being non-existent, the vast majority of patients report on a previous cutaneous squamous cell carcinoma (CSCC) in the head and neck area [10, 11], typically 1 year after onset of disease [12]. Therefore, these parotid tumours are in fact representing CSCC-derived lymph node metastases [13]. Eighty percent of all CSCC are found in the head and neck region [14]. High exposure to ultraviolet (UV) and ionising radiation as found in Australia was reported to foster the formation of CSCC [11].

The objective of our study was to analyse and compare the clinical behaviour of primary PGC and CSCC metastatic parotid cancers with special emphasis on therapy and oncologic outcome.

## Methods

All patients with histologically proven malignant tumours of the parotid gland who underwent combined surgery and radiation therapy or surgery alone at the Department of Otorhinolaryngology, Head and Neck Surgery of the University Hospital Cologne, Germany, between January 1987 and December 2015 were retrospectively assessed thus identifying all cases of metastatic parotid CSCC. Clinical data were retrieved from patients’ medical records, histology reports, and radiographic imaging. TNM staging was performed according to the 8th edition of the American Joint Committee on Cancer (AJCC) [15]. Demographic data as well as oncological outcomes were compared between metastatic CSCC of the parotid gland and primary parotid gland tumours.

### Therapy

All clinical cases had been discussed at a multidisciplinary tumour board meeting prior to treatment. Before surgery, a fine needle aspiration of the mass was performed. In case of suspected malignancy, an intraoperative frozen section procedure was performed and surgery was extended to a total or radical parotidectomy and neck dissection. Patients with clinically and radiologically negative neck nodes were treated with selective neck dissection level [16, 17]. Preoperative clinical facial nerve palsy and obvious tumour infiltration of the facial nerve intraoperatively resulted in resection of the facial nerve and reconstruction in selected cases.

Additional adjuvant radiation therapy was indicated in cases of high-grade carcinoma (G3 or G4), adenoid cystic carcinoma, positive resection margins, cervical lymph node metastasis, and perineural invasion. These patients received a daily fraction of 1.8–2.0 Gy five times a week by a linear accelerator (LINAC, 6 MV-photons). The ipsilateral cervical lymph node levels (levels I–V) received 50 Gy while the parotid gland region and tumour affected levels of the neck have been irradiated with 60–65 Gy.

All patients underwent regular follow-up examinations every 3 months in the first year, every 6 months for the subsequent 3 years, and annually from the fourth year onward. Residents’ registration offices were consulted for information regarding residential status or death.

### Statistical analysis

The overall survival rates were assessed using the Kaplan-Meier method for incomplete observations. The log-rank test was then used to detect correlations between prognostic factors and outcome. A *p* value of < 0.05 was considered statistically significant. All statistical tests were performed using SPSS (IBM SPSS Statistics 25.0, IBM, New York City, NW, USA).

## Results

A total of 342 patients suffering from malignant tumours of the parotid gland were identified. Forty-nine out of these were diagnosed with metastatic CSCC of the parotid gland.

### Primary parotid gland carcinomas

**Fig. 1:**
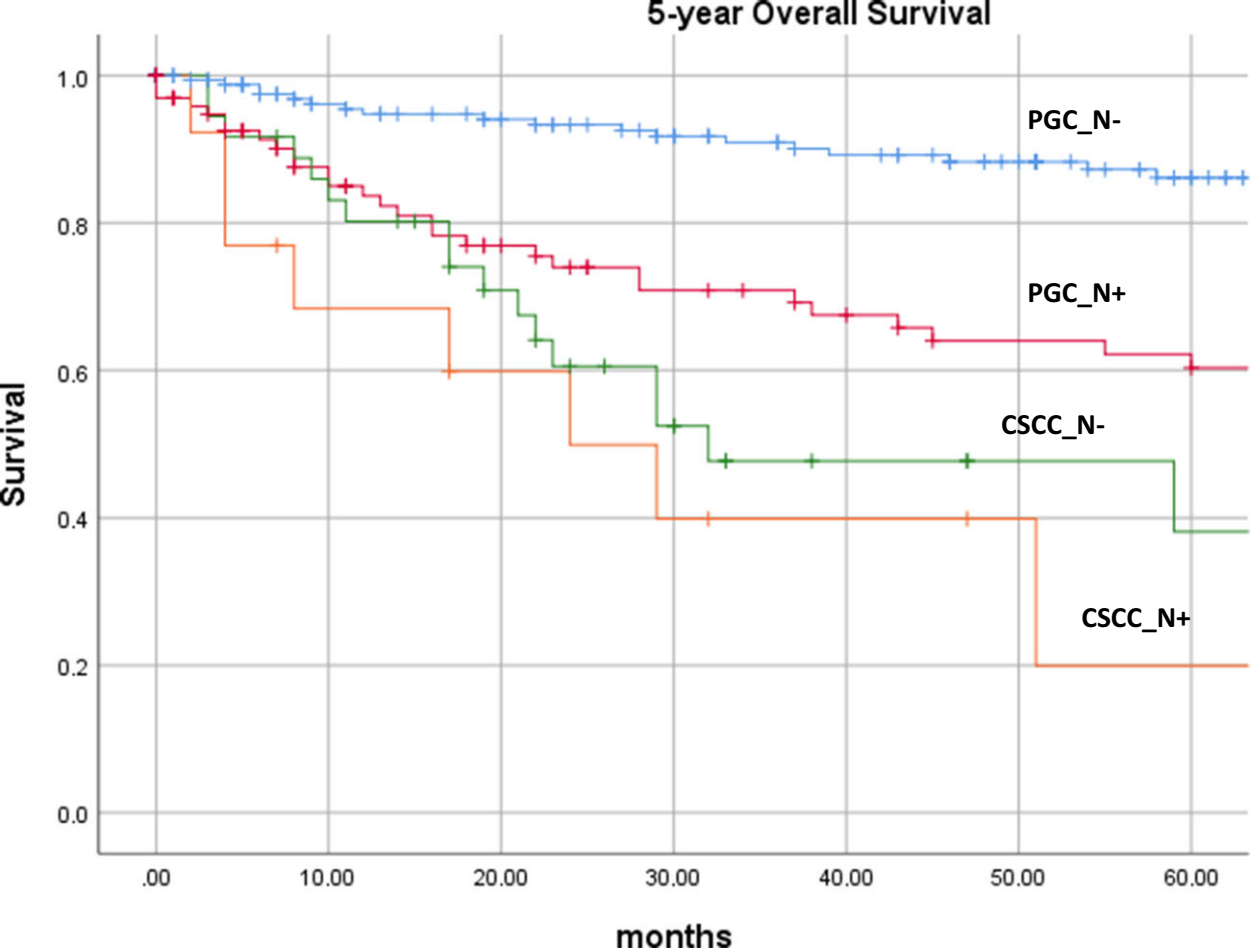
Five-year overall survival rates calculated by the Kaplan-Meier method of the primary PGC patients’ cohort with (PGC_N+) and without (PGC_N-) positive lymph nodes in the neck, as well as CSCC patients’ cohort with (CSCC_N+) and without (CSCC_N-) lymph node metastasis. The 5-year overall survival in PGC was 60.3% (N+) and 86.1% (N-), and 19.9% (N+) and 38.1% (N-) in CSCC, respectively. No significant survival difference could be detected between patients with sole involvement of the parotid gland (CSCC_N-) compared to patients with additional neck lymph nodes CSCC_N+ (*p* = 0.109). Even the unfavourable group of PGC with positive neck lymph nodes (PGC_N+) showed a significantly better prognosis as compared to CSCC without additional cervical lymph nodes (CSCC_N-) (*p* = 0.008)

The remaining 293 patients with primary malignomas of the parotid gland yielded a mean age of 56.8 years (7–91 years) and male to female ratio of 1:1 (Table 1). Histology was adenocarcinoma NOS (*n* = 56), mucoepidermoid carcinoma (*n* = 48), adenoid cystic carcinoma (*n* = 45), acinic cell carcinoma (*n* = 41), epithelial-myoepithelial carcinoma (*n* = 15), carcinoma ex pleomorphic adenoma (*n* = 14), undifferentiated carcinoma (*n* = 15), salivary duct carcinoma (*n* = 9), basal cell adenocarcinoma (*n* = 9), and other rare entities (*n* = 41). Rate of lymph node metastases was 27.3%; infiltration of the facial nerve was reported in 15.4%. A total of 45.1% received postsurgical adjuvant radiation therapy. Five-year overall survival was 77.2% in all primary PGC patients (Table 1). In case of histologically proven loco-regional lymph node metastasis (PGC_N+), 5-year overall survival rate declined from 86.1 (N0 neck) to 60.3% (Fig. 1) (*p* < 0.001).

**Table 1:** Demographic data

	Parotid metastases of SCCS	Primary parotid gland carcinoma
Number of patients	49	293
Mean age in years (min–max)	72.3 (30–93)	56.8 (7–91)
Sex		
Female	16.3%	51.2%
Male	83.7%	48.8%
Nodal involvement (N+)	100%	27.3%
Infiltration of facial nerve or skin	42.9%	15.4%
Operative therapy	100%	100%
Radiation therapy	93.9%	45.1%
5-year overall survival	32.6%	77.2%

### CSCC

**Fig. 2:**
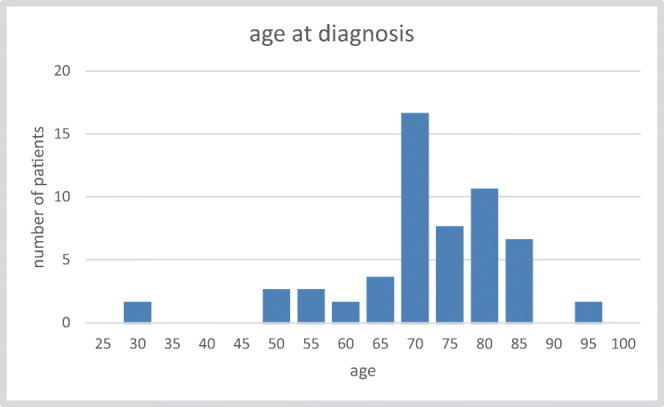
Age distribution of CSCC patients

Mean age for CSCC patients (*n* = 49) was 72.3 years (30–93 years) (Fig. 2) with a male to female ratio of 5:1. The age of CSCC patients was thus significantly higher than the age of patients with PGC (*p* = 0.012). Table 1 depicts the clinical data including the type of therapy. Of note, six patients who underwent a lateral parotidectomy refused any extended tumour surgery. Three patients refused a further adjuvant therapy.

**Fig. 3:**
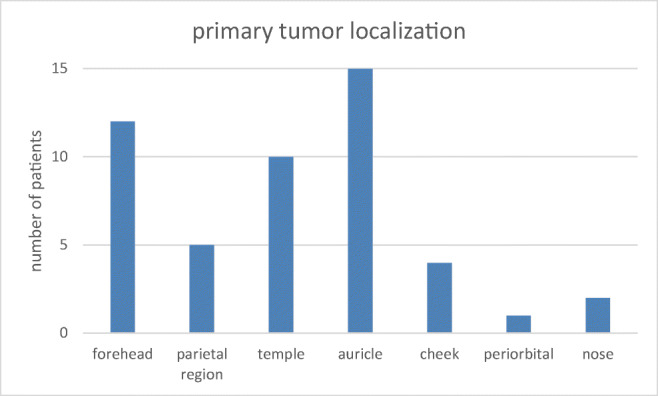
Origin of parotid gland metastasised CSCC

Primary CSCC tumours were located at the forehead (*n* = 12), parietal region (*n* = 5), temple (*n* = 10), auricle (*n* = 15), cheek (*n* = 4), periorbital region (*n* = 1), and nose (*n* = 2) (Fig. 3).

Mean follow-up was 31 months. Five-year overall survival rate was 32.6%, i.e. yielding a significantly worse outcome as compared to PGC patients irrespective of lymph node metastasis (*p* < 0.001). No significant survival difference could be detected between patients with sole involvement of the parotid gland (CSCC_N-) compared to patients with additional neck lymph nodes CSCC_N+ (*p* = 0.109). Nevertheless, 19.9% 5-year overall survival in the group of patients with additional lymph node metastases (CSCC_N+) was even less favourable as compared to patients with only parotid gland metastasis(s) (CSCC_N-) with an overall survival of 38.1%. Even the unfavourable group of PGC with positive neck lymph nodes (PGC_N+) showed a significantly better prognosis as compared to CSCC without additional cervical lymph nodes (CSCC_N-) (*p* = 0.008) (Fig. 1).

## Discussion

In contrast to other previously published studies, this study focuses on malignancies of the parotid gland and distinguishes between primary and secondary tumours with respect to clinical and therapeutic characteristics as well as 5-year overall survival. PGC were mainly classified as adenocarcinoma NOS, mucoepidermoid, adenoid cystic, and acinic cell carcinoma. A total of 77.2% 5-year overall survival rate is comparable to previously published results [18, 19].

In the CSCC group, the majority of patients were male. This is consistent with already published data of PGC [20]. The age distribution of the CSCC patients with parotid involvement presented here also agrees with data from previously published patient cohorts thus confirming that older patients are particularly affected by that disease [20].

Primary CSCC were most often located in the area of the auricle, temple, and forehead. This is in accordance with previous reports [12, 21]. Creighton and colleagues showed that CSCC preferentially metastasise to the forehead (85%), periauricular area (76%), and in 30% to the scalp, cheek, and infraauricular region [21]. Hirshoren et al. further demonstrated that the majority of CSCC originating from the scalp, auricle, and cheek area metastasise to the parotid gland [12].

Despite multimodal therapeutic strategies, the 5-year OS remained poor in CSCC patients (32.6%) as compared to PGC (77.2%). These results are in line with previously published data of other authors [11, 20, 22] and are due to a generally higher tumour stadium as a consequence of lymph node metastasis in the CSCC group. It is noteworthy that even PGC patients having loco-regional metastasis had a better 5-year OS as compared to CSCC patients irrespective of neck node metastasis (CSCC_N- and CSCC_N+). Cervical metastases were demonstrated to significantly worsen the prognosis of CSCC patients [11, 20]. However, in our study, we could not find a significant difference in 5-year overall survival for CSCC patients without further neck lymph node metastases (CSCC_N-) compared to CSCC with neck lymph node metastases (CSCC_N+).

It should be discussed how the overall survival in this group could be improved: On the one hand, studies indicate that an improvement in diagnosis and consistent implementation of adequate staging and timely initiation of therapy can improve overall survival. Deilhes et al. demonstrated that 37% of patients were not diagnosed until the disease was in an advanced stage, indicating a lack of CSCC identification. For the remaining 69 patients, 7% did not receive treatment within 3 months of the CSCC being identified, 62% had an incomplete histological report, and 37% had incomplete treatment [23]. On the other hand, an escalation of therapy in order to improve overall survival seems reasonable. But at least, all patients with advanced CSCC, like in our study, had received both radical surgery as well as adjuvant radiotherapy. Increasing the radicality of the surgery might lead to a better survival. Coombs et al. concluded that more extensive surgery, including lateral temporal bone resection, could improve the local control rate in cases of advanced disease [24]. For better overall survival, immunotherapy might also be added to standard therapy in an adjuvant or neoadjuvant setting in the future. Current drug therapy options were examined in a palliative setting by several authors. Montaudie et al reported on cetuximab as monomodal therapeutic option in unresectable palliative CSCC patients (*n* = 58, mean age 83.2 years) [25]. The overall response rate (ORR) was 53% and 42% after six and 12 weeks, respectively. The authors conclude that cetuximab delays disease progression [25]. In a review by de Lima et al., the authors summarised studies on CSCC drug therapy. Again, the application of cetuximab was discussed in combination with checkpoint inhibitors [26]. Checkpoint inhibitors could serve as a therapeutic alternative in case of recurrent CSCC yielding parotideal metastases. Compared to platinum-based chemotherapy, modern immunotherapeutic strategies are considered as being better tolerated especially in elderly patients. Recently, the PD-1-blocking antibody cemiplimab was approved by the FDA and EMA for advanced CSCC treatment. However, detailed guidelines for indication are still missing which might be—at least in part—due to a lack of appropriate clinical studies for patients with recurrent or metastasised CSCC [27]. Steeb et al. reviewed the previous studies and experiences using checkpoint inhibitors in advanced CSCC and concluded that cemiplimab and pembrolizumab immunotherapy could result in a response rate of 40–55% in a first-line palliative setting [27–29]. These promising results might be due to a high immunogenicity of CSCC [30]. However, the exact setting or composition in which immunotherapy should be applied remains a matter of debate.

The retrospective character of our study and potentially associated selection bias as well as the relatively low number of patients with CSCC limits clinical validity.

## Conclusions

The present study retrospectively evaluated 342 patients with primary PGC (*n* = 293) and CSCC metastatic cancer to the parotid gland (*n* = 49) thus yielding a significantly worse prognosis for metastasised CSCC despite an intense multimodal therapeutic effort (radical surgery and adjuvant radiotherapy.

## Data Availability

All data are available on request from the corresponding author.
